# Genome-wide Identification and characterization of circular RNAs in the rice blast fungus *Magnaporthe oryzae*

**DOI:** 10.1038/s41598-018-25242-w

**Published:** 2018-04-30

**Authors:** Jialan Yuan, Zhao Wang, Junjie Xing, Qingyong Yang, Xiao-Lin Chen

**Affiliations:** 1 The Provincial Key Lab of Plant Pathology of Hubei Province, College of Plant Science and Technology, Huazhong Agricultural University, Wuhan, Hubei China; 2 College of Informatics, Hubei Key Laboratory of Agricultural Bioinformatics, Huazhong Agricultural University, Wuhan, 430070 China; 3 State Key Laboratory of Hybrid Rice, Hunan Hybrid Rice Research Center, Changsha, Hunan China

## Abstract

Numerous circRNAs have been identified in different organisms, but little attention has been addressed on fungal circRNAs. Here, we identified a total of 8,848 circRNAs from the model plant pathogenic fungus *M. oryzae*. 5,840 circRNAs were identified from mycelium, 2,721 circRNAs from conidium, while only 287 circRNAs from both tissues. This indicated that most of the *M. oryzae* circRNAs were specifically expressed in mycelium or in conidium. Parental genes of circRNAs in mycelium were enriched in basic metabolisms required for normal growth, while in conidium, they were enriched in biogenesis of storages potentially used for infection. *M. oryzae* circRNAs could also bind to miRNAs, suggesting they may also function as sponges in fungi. This study suggested *M. oryzae* circRNAs could play important roles in regulation of growth and development.

## Introduction

Non-coding RNAs (ncRNAs) have been found to be widely distributed in eukaryotic cells and play important roles in distinct biological processes^1^. Circular RNAs (circRNAs) are one type of ncRNAs, which were rarely identified before, but since several years ago, by using new generation RNA sequencing technologies together with bioinformatics tools, thousands of circRNAs were identified across different species, such as plant, mammal, and human^2–5^.

CircRNAs are characterized by closed continuous loop structures with covalently linked 3′ and 5′ ends, in contrast to the linear RNAs structures with 5′ caps and 3′ tails. As a result, the circRNAs can’t be digested by RNase R, a 3′ to 5′ exoribonuclease which can efficiently degrade linear RNAs. CircRNAs can be generated by back-splicing, a type of variant splicing which leads to circularize the 5′ end of the upstream exon to the 3′ end of the downstream exon^5^, it can also be generated by other mechanisms from both introns and intergenic regions^6–8^.

At present, the general functions of circRNAs have not been well uncovered. One of the well described roles of circRNAs is acting as miRNA sponges to inhibit functions of miRNAs^2,9,10^. In human, the circRNA ciRS-7 was firstly found as a miRNA sponge to efficiently bind miR-7 and suppress its activity, and then increase expression of its target genes^9^. With different mechanisms, circRNAs may act as transcriptional regulators to regulate different biological processes. In plant, for example, circRNAs have been reported to be involved in response to abiotic stresses such as Pi starvation and chilling^5,11,12^.

Since first discovered in human^13^, up to now, genome-wide identification of circRNAs has also been performed in Archaea^14^, *Caenorhabditis elegans*^15^, mice^10^, and plants^5^. According to those studies, the expression patterns of circRNAs were specific in different development stages and environmental conditions, tissues, and cell types in kinds of organisms. Compared with more attention to those mentioned organisms, genome-wide characterization of circRNAs in fungi has not been reported.

*Magnaporthe oryzae* is a model plant fungal pathogen which is a serious threat to rice production worldwide^16,17^. This fungus can produce a great deal of conidia, by which it forms specialized appressoria on the host surface for penetration and infection in the host cells^16,17^. In the present study, we use *M. oryzae* as a model to genome-widely identify circRNAs in fungi. We systematically analyzed the circRNAs from mycelium and conidium, by using high-throughput sequencing technology and bioinformatic tools. A total of 8848 circRNAs were identified and characterized from these two samples. Combined with the bioinformatics analyses, our results demonstrated different expression patterns and roles of circRNAs in distinct developmental processes.

## Results

### RNA sequencing and identification of circRNAs in *M. oryzae*

In order to genome-widely identify circRNAs in *M. oryzae*, total RNAs were extracted from mycelium and conidium. Two biological replicates of each tissue were used, designated with MY_1, MY_2, CO_1, and CO_2, respectively. Figure S1 showed flowchart for identification of circRNA in *M. oryzae*. Total RNAs of each samples was conducted with rRNA depletion and RNase R treatment, and the remaining RNAs were used to construct libraries, which were sequenced by an Illumina Hiseq. 2500 analyzer. When adaptor sequences and low-quality reads were removed, the clean reads were subjected by find_circ software to identify circRNAs. A set of confident back-spliced junction reads including 702 from MY_1, 2, 517 from MY_2, 2,386 from CO_1, and 4,068 from CO_2, were obtained for identifying circRNAs (Table S1).

### Properties of circRNAs in *M. oryzae*

**Figure 1:**
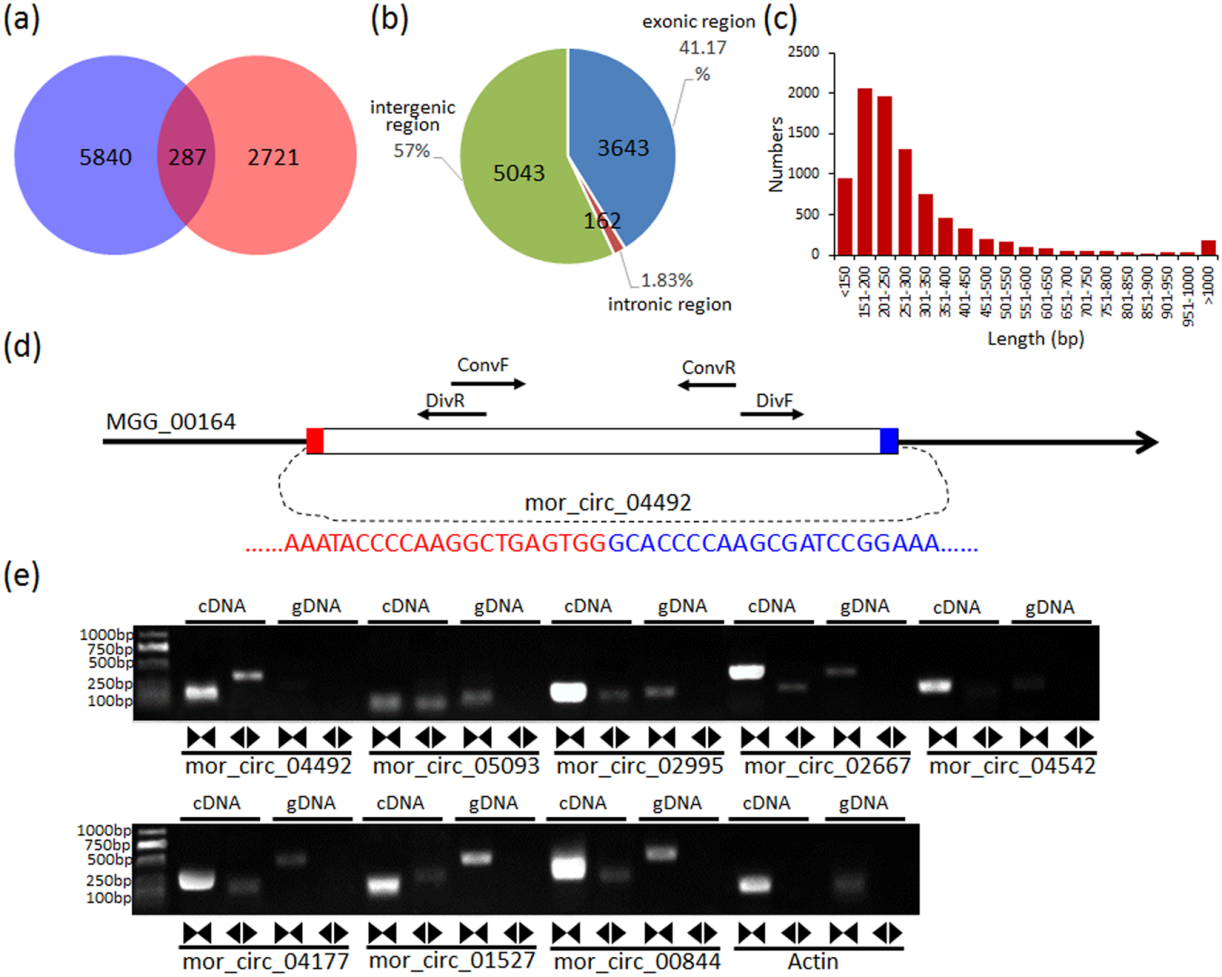
Sequence features and validation of circRNAs in *M. oryzae*. (**a**) Venn diagram showing the number of tissue-preferentially expressed circRNAs in mycelium and conidium of *M.oryzae*. (**b**) Distribution of circRNAs in genome region of *M. oryzae*. (**c**) Length distribution of circRNAs. (**d**) An example of *M. oryzae* circRNAs (mor_circ_04492) shows the validation strategy. Divergent and convergent primers were designed to detect circular RNAs. Sanger sequencing was performed to confirm head-to-tail backsplicing. (**e**) Experimental validation of *M. oryzae* circRNAs. Divergent primers successfully amplified circRNAs in cDNA but failed in genomic DNA. Amplification for sequence of actin gene was used as a control. The gels were cropped from the same gel, and the full-length gel was supported in Fig. S2.

In total, we identified 8,848 novel circRNAs (Dataset S1), whose sequences were shown in Dataset S2. Among these circRNAs, 5,840 were identified from mycelium, 2,721 were identified from conidium, and only 287 were identified from both samples (Fig. 1a). In total identified circRNAs, 3,643 (41.17%) were generated from exonic regions, 162 (1.83%) were from intronic regions, and 5,043 (57.00%) were from intergenic regions (Fig. 1b). These circRNAs distributed in 61 scaffolds among total 219 scaffolds in genome of the P131 strain, and P131_scaf1.3 contains the most numbers of circRNAs (1,561, 17.64%) (Table S2). The length of circRNAs were mostly between 150 bp and 500 bp, while only a few circRNAs were more than 1000 bp (Fig. 1c).

### Validation of *M. oryzae* circRNAs

We then experimentally validated the predictions of circRNA in *M. oryzae*. We randomly selected ten circRNAs from the highly expressed ones for validation. These selected circRNAs were validated by reverse transcription (RT)-PCR method. We designed divergent and convergent primers to amplify each circRNA in the total RNA and genomic DNA (Table S3). Theoretically, divergent primers could amplify the fragment from circular form in RNA samples, but convergent primers couldn’t amplify any sequence. The divergent primers also couldn’t amplified target fragments in genomic DNA. The PCR products were subsequently analyzed by agarose gel electrophoresis and verified by sequencing (Figs 1d,e and S2). Eight circRNAs (80%) were successfully validated, indicated the identified circRNAs in this study were reliable.

### Expression patterns of circRNAs in *M. oryzae*

**Figure 2:**
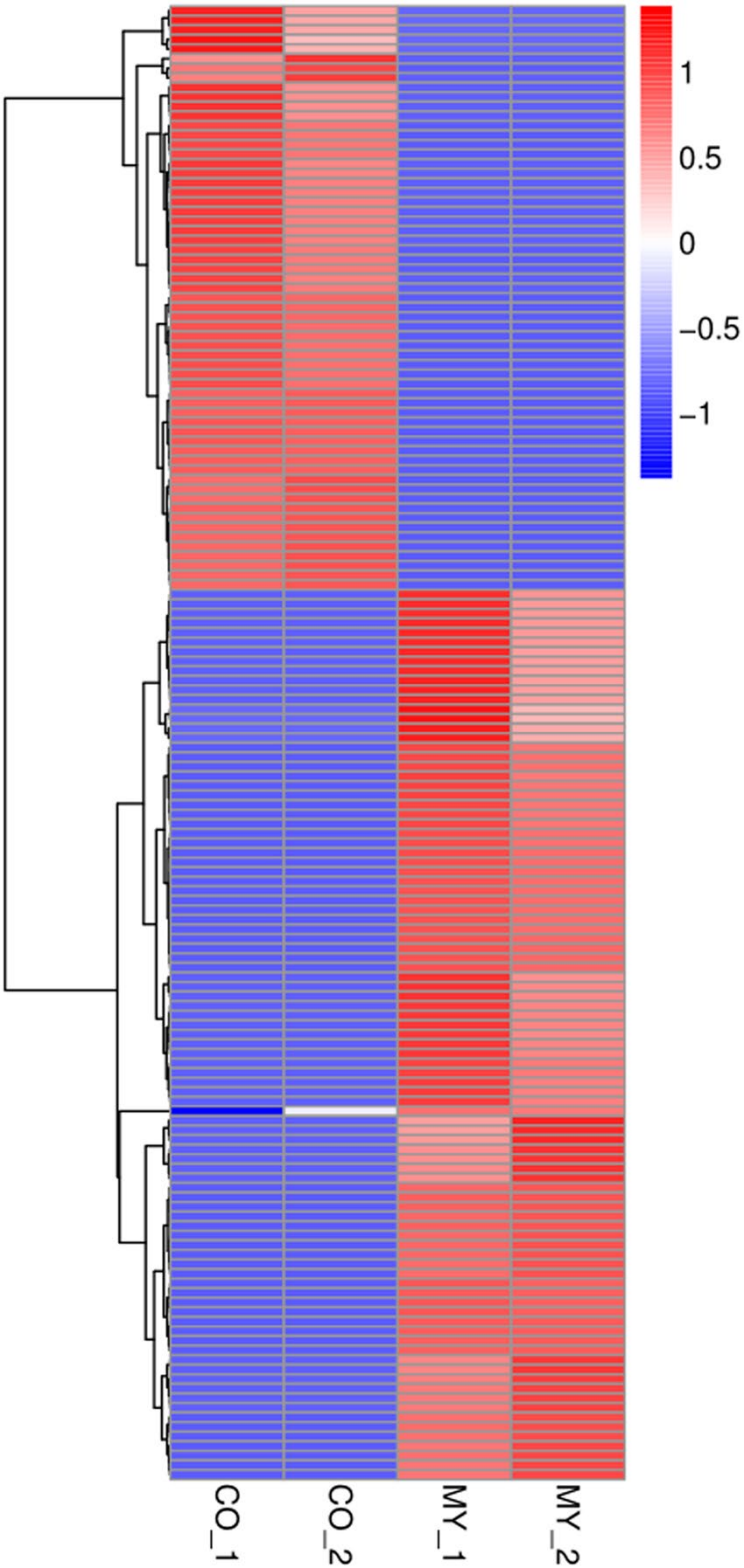
Heatmap showing the expression patterns for all the circRNAs identified in *M. oryzae*. Vertical columns represented different tissues of *M. oryzae*. Horizontal rows represented circRNAs. Color scale representing Z-score was given at the left.

In order to reveal expression patterns in different developmental stages, the expression levels of all circRNAs were normalized by using a TPM (transcripts per million) analysis^18^. In total, approximately 95.3% (5840) of mycelium circRNAs and 90.5% (2,721) of conidium circRNAs were tissue specific, but only 2.7% (287) of the total circRNAs were commonly expressed in both tissues (Fig. 1a). Hierarchical cluster analysis also revealed circRNAs exhibited specific expression patterns in mycelium and conidium of *M. oryzae* (Fig. 2).

### Functional classification of circRNAs

To explore putative functions of circRNAs in different developmental stages of *M. oryzae*, the parental genes of circRNAs expressed in mycelium or conidium were conducted by Gene Ontology (GO) classification and Kyoto Encyclopedia of Genes and Genomes (KEGG) pathway analysis, respectively.

**Figure 3:**
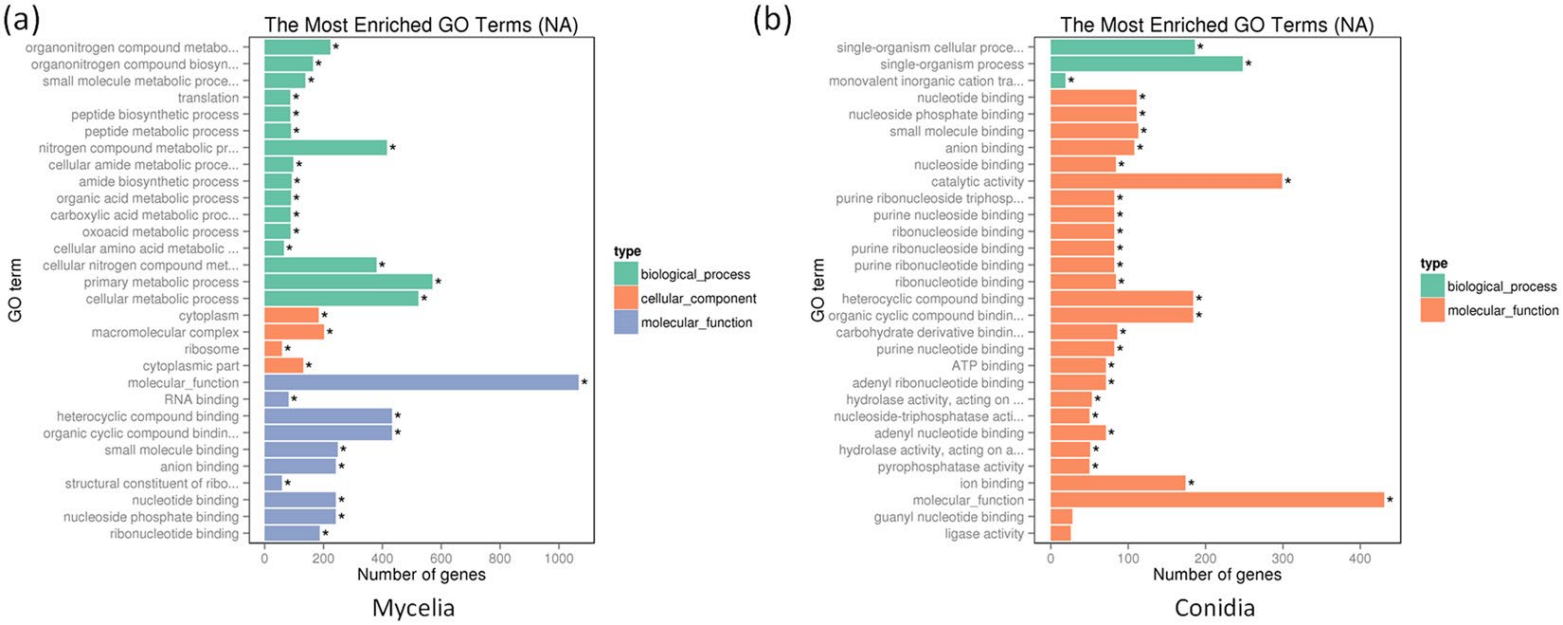
GO categories of circRNA-host genes in mycelium (**a**) and conidium (**b**) of *M. oryzae*.

**Figure 4:**
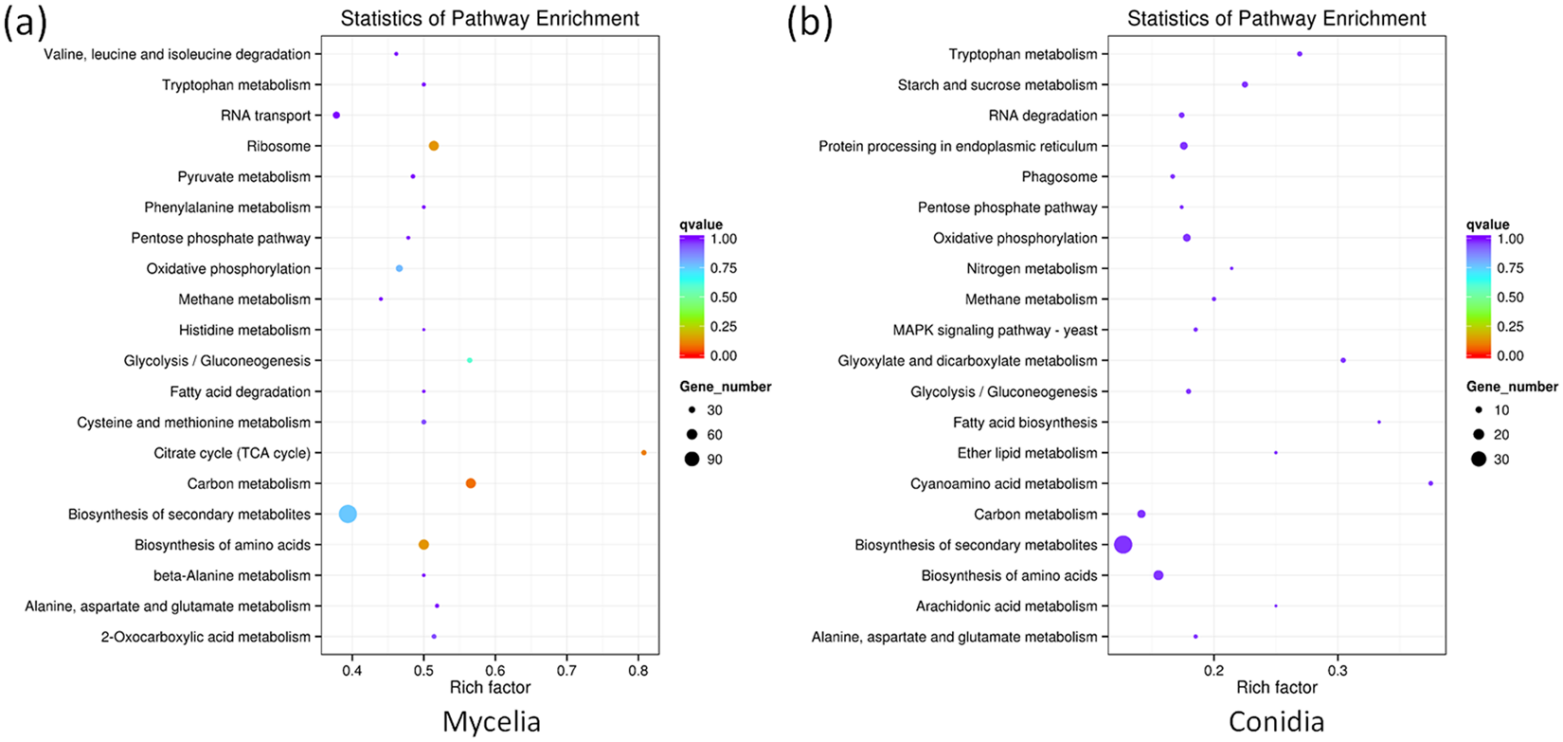
KEGG pathway analysis of circRNA-host genes in mycelium (**a**) and conidium (**b**) of *M. oryzae*.

In mycelium, among the biological process classification, the enriched GO terms included single-organism cellular process (GO:0044699), cellular process (GO:0009987), and metabolism process (GO:0008152) such as cellular metabolism process (GO:0044237), nitrogen compound process (GO:0006807) and basic metabolism process (GO:0044238). In the molecular function classification, the circRNA-host genes were mainly related to ribosome component (GO:0005198) and ribosome binding (GO:0005488), including heterocyclic compound binding (GO:1901363), organic cyclic compound binding (GO:0097159), small molecule binding (GO:0036094), nucleotide binding (GO:0000166) and RNA binding (GO:0003723), etc. For cellular component classification, the circRNA-host genes encoded proteins were mainly located in macromolecule complex (GO:0032991), cytoplasm (GO:0005737), cytoplasmic part (GO:0044444), and ribosome (GO:0005840) (Figs 3a and S3). The KEGG pathway analysis demonstrated the circRNA-host genes in mycelium were significantly enriched in amino acid metabolism (such as tryptophan metabolism, alanine, aspartate and glutamate metabolism, etc.), sugar metabolism, carbon metabolism, and methane metabolism. On the other hand, some biosynthesis pathways, such as biosynthesis of amino acids and secondary metabolisms, as well as the ribosome structure formation were also enriched (Fig. 4a; Table S4).

In conidium, for the biological process, the enriched GO terms included single-organism process (GO:0044699) and single-organism cellular process (GO:0044763). For the molecular function, the circRNA-host genes were mainly related to catalytic activity (GO:0003824) and binding (GO:0005488), such as heterocyclic compound binding (GO:1901363), organic cyclic compound binding (GO:0097159), ion binding (GO:0043167), and small molecule binding (GO:0036094). Interestingly, many nucleoside, nucleotide, ribonucleoside and ribonucleotide binding activities were found, indicated circRNA could play key roles in regulation of nuclear events in conidium (Figs 3b and S4). The KEGG pathway analysis demonstrated the circRNA-host genes in mycelium were significantly enriched in amino acid metabolism (such as tryptophan metabolism, alanine, aspartate and glutamate metabolism, etc.), sugar metabolism, lipid metabolism and pentose phosphate pathway. For biosynthesis, fatty acid biosynthesis, biosynthesis of amino acids, biosynthesis of secondary metabolites were also enriched. For structure formation, phagosome and protein processing in endoplasmic reticulum were enriched (Fig. 4b; Table S5).

The GO category and KEGG pathway analyses showed that, in mycelium, host genes of circRNAs were mainly involved in basic metabolisms, which were required for normal growth. While in conidium, circRNAs host genes were mainly involved in biogenesis of different storages, which could be used for the appressorium-mediated fungal infection. These data suggested the circRNAs could play important roles in growth and development in *M. oryzae*.

### circRNA-miRNA interaction analysis

**Figure 5:**
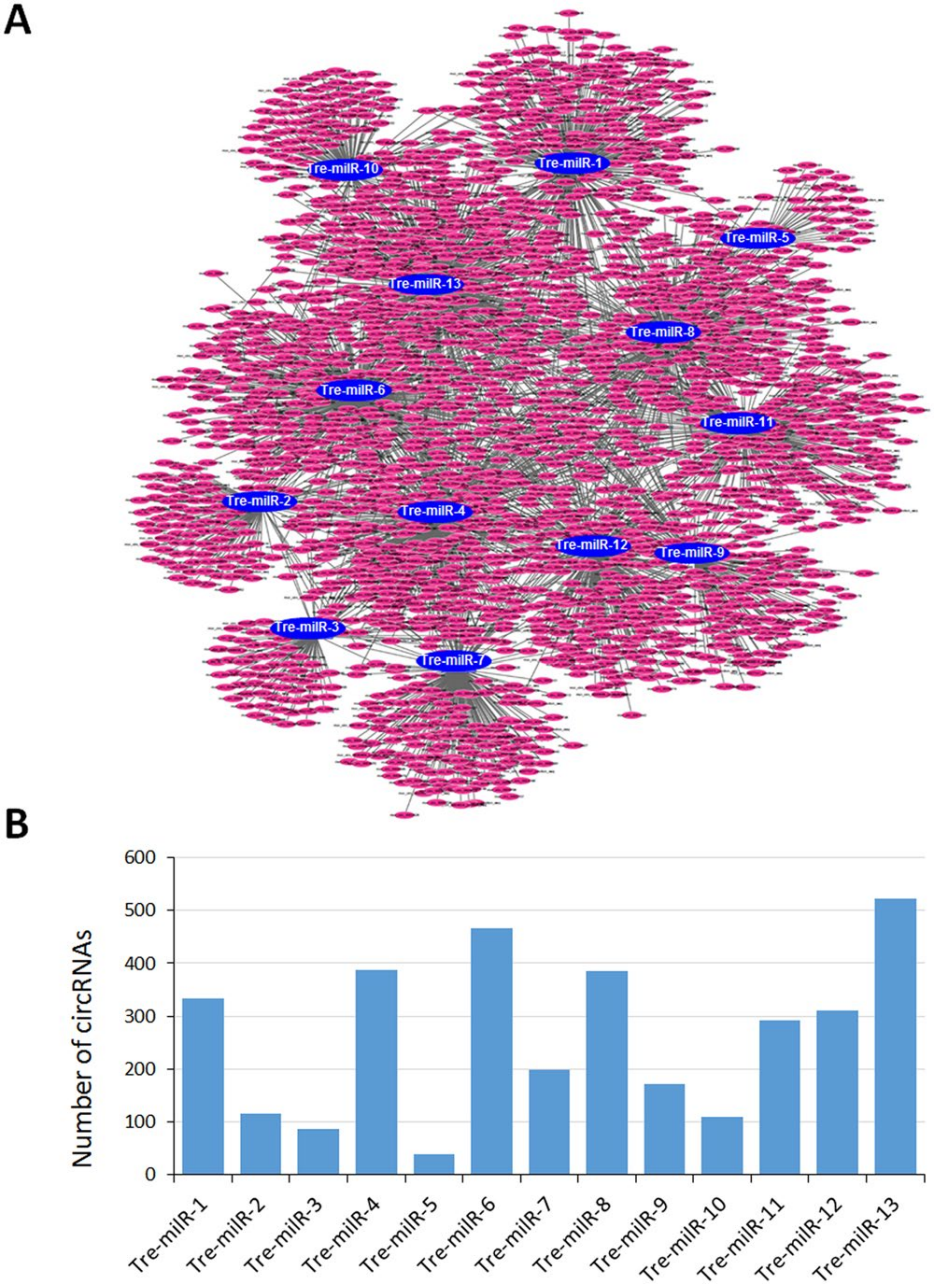
CircRNA-miRNA interaction network in *M. oryzae*.

It has been widely reported that circRNAs can bind miRNAs to prevent them from targeting mRNAs and then activate gene expression^8^. To determine whether *M. oryzae* circRNAs could affect post-transcriptional regulation of function genes by binding to miRNAs, all the circRNAs sequences were used to analyze potential miRNAs binding sites. Because the *M. oryzae* miRNA has not been reported yet, we used the published miRNAs identified in a filamentous fungus *Trichoderma reesei*, as potential circRNAs targets. In total, we identified 2,760 (31.19%) circRNAs contained predicted binding sites for 13 miRNAs, and 534 of them contain two to eight miRNA binding sites (Dataset S3). Through Cytoscape software, we construct an entire circRNA-miRNA interaction network based on the interaction predicted by conserved seed-matching sequence between circRNAs and miRNAs (Fig. 5A). We found that Tre-milR-13, Tre-milR-6, and Tre-milR-4 could be bound by 552, 467, and 388 circRNAs, respectively (Fig. 5B). On the other hand, for example, miRNAs Tre-milR-6, Tre-milR-8 and Tre-milR-12 could all be bound by the circRNA mor_circ_01113 (Dataset S3). These interactions indicated that not only one miRNA could be bound by different circRNAs, but also one circRNA could bind different miRNAs. These data suggested the circRNAs could also function as miRNAs sponges to regulate gene expression in *M. oryzae*.

## Discussion

In the past several years, thousands of circRNAs were identified in humans, animals, plants (including *Arabidopsis*, rice, and soybean), yeasts (*S. cerevisiae* and *Schizosaccharomyces pombe*)^5,10,14,15,19^, and the numbers are growing quickly. Because the circRNAs are largely distributed in different eukaryotic organisms, and their features are quite unique, they could function as important post-transcriptional regulators, which need more extensive research. However, except for yeasts, genome-wide and systematic identification of fungal circRNAs has not been reported so far and their features are largely unknown. In this study we conducted a genome-wide identification of circRNAs in the model plant pathogenic fungus *M. oryzae*, and analyzed the features of circRNAs in this fungus. Our results demonstrated a large number of circRNAs exist in the fungus. Our data also provide resources for further functional characterization of *M. oryzae* circRNAs.

In the identified *M. oryzae* circRNAs, 5840 (66.00%) were specifically derived from mycelium, and 2721 (30.75%) were specifically derived from conidium (Fig. 1A). It seems like that, the numbers of circRNAs in mycelium may be larger than that in conidium, suggesting more unique circRNAs could be required for regulation in mycelium formation. Since only the mycelium and conidium tissues were used to identify circRNAs, the actual number of circRNAs in *M. oryzae* should be definitely underestimated.

Similar to humans, animals, and plants^3–5^, majority of the expression profiles of *M. oryzae* circRNAs were specifically expressed in different tissues (Fig. 2). In 8,848 identified circRNAs, only 287 (3.24%) were identified in both of the mycelium and conidium. This phenomenon suggested that specific developmental stage could be regulated by corresponding circRNAs. More interestingly, the GO category and KEGG pathway analyses showed that, in mycelium, parental genes of circRNAs were mainly involved in basic metabolisms related to normal growth. While in conidium, parental genes of circRNAs were basically involved in biogenesis of different storages (especially glycogen, lipid, and kinds of amino acids), which could be utilized for the appressorium-mediated fungal infection process. These data suggested the circRNAs could play important roles in mycelium and conidium development in *M. oryzae*. However, it is more interesting to evaluate functions of circRNAs during appressorium formation and invasive growth in the host cells.

We also identified 2,760 (31.2%) circRNAs contained potential binding sites for 13 miRNAs target mimics in fungus^20^. These circRNAs-miRNA interactions could function as potential post-transcriptional regulators like miRNA sponges. However, the binding events and biological functions of circRNAs-miRNA interactions need experimental validation. On the other hand, no miRNA binding sites can be predicted in other 68.8% circRNAs, what regulatory mechanisms existed in these circRNAs should be addresses in the future.

In this study, by using high-throughput RNA sequencing technology and bioinformatics tools, we were able to identify and characterize the *M. oryzae* circRNAs from mycelium and conidium. We analyzed their features, expression patterns and potential functions. In the future, it is required to explore and compare circRNAs from fungal appressorium formation stage and infection hyphae formation stage. Expression patterns of circRNAs under distinct stresses also need research. Importantly, determine roles and regulatory mechanisms of key circRNAs, especially the pathogenicity-related circRNAs, are required. For example, whether *M. oryzae* circRNAs can bind miRNAs from itself or host is an interesting area. Our study not only reveals the features of circRNAs in fungi, but also provided resource of circRNAs for further study, such as the regulatory mechanisms and evolution of circRNAs in different organisms.

## Methods

### Materials preparation

The *M. oryzae* strain P131 was used for test^21^. Mycelium were collected from two-day-old culture in liquid CM at 180 rpm (28 °C). Conidia were washed down from colony grown on seven-day-old oatmeal agar plates and were harvested by filtering through five layers of lens wiping paper.

### Libraries construction for circRNA sequencing

The total RNAs were isolated from mycelium and conidium tissues by using TRIzol reagent (Invitrogen, CA, USA) according to the manufacturer’s procedure. Concentration and purity of the total RNA were detected with a NanoDrop spectrophotometer ND-1000 (NanoDrop Technologies, Wilmington, DE, USA). Around 10 µg total RNA was used for removing of rRNA by the Epicentre Ribo-Zero Gold Kit (Illumina, San Diego, USA). Next, the RNAs were treated with 10 U/μg RNase R (Epicentre, Madison, WI) to remove linear RNA at 37 °C for one hour. By using an mRNA-Seq sample preparation kit (Illumina, San Diego, USA), the remaining RNAs were used to construct cDNA libraries, which were subsequently used to perform circRNA sequencing by an Illumina Hiseq2500 platform.

### Identification of circRNAs in *M.oryzae*

*M. oryzae* genome sequence and gene annotations of P131 strain were downloaded from NCBI Genome Database (www.ncbi.nlm.nih.gov/genome) under the accession number AHZT00000000^22^. Adapter sequences and low quality reads were removed from the raw reads. Then the high-quality clean reads were used for circRNAs identification according to pipline described in Fig. S1. In brief, index of the reference genome was built using Bowtie (v2.0.6)^23^, and paired-end clean reads were multiply mapped with the *M.oryzae* reference genome through Tophat2 (v2.1.0) algorithm^24^. The unmapped reads were kept, and 20-mers sequences from 5′ and 3′ end of these reads were used to align reference genome again, by using a software Bowtie v2.0.6^22^. The anchored sequences were subsequently analyzed by the find_circ software^2^, then the complete reads can be aligned with breakpoints flanked by GU/AG splice sites. At last, back-spliced reads with more than two supporting reads were identified as circRNAs.

### Validation of circRNAs in *M. oryzae*

Genomic DNA was extracted from mycelium and conidium tissues by the cetyl-trimethylammonium bromide (CTAB) method^25^. Total RNA was isolated from mycelium and conidium tissues using TRIzol reagent (Invitrogen, CA, USA) according to the manufacturer’s method. Total RNA samples were treated with DNase I to remove DNA contamination (Takara, Dalian, China). The cDNA were obtained by reverse transcription using PrimeScript RT-PCR Kit (Takara, Dalian, China) with random primers. The divergent and convergent PCR primers were designed for circRNA validation. Divergent primers were designed on the flanking sequences of head-to-tail splicing sites of circRNAs to detect the circRNAs (Table S3). Convergent primers were used as positive controls to detect linear transcripts. Divergent PCR amplification were performed by 36 cycles, while convergent PCR amplification were performed by 26 cycles. The PCR products were dissected by gel electrophoresis and purified by the QIA quick Gel Extraction Kit (Qiagen, Beijing, China). Sanger sequencing was performed subsequently.

### Expression level analysis

To reveal expression patterns in distinct developmental stages, the expression levels of all circRNAs were normalized by using TPM (transcripts per million) algorithm^18^: Normalized expression = (mapped reads)/(total reads) * 1000000. Differential expression of two samples was performed by using DESeq. 2^26^. P-values was adjusted by Benjamini & Hochberg method^27^. By default, the threshold of corrected p-value for differential expression was set to 0.05.

### GO categories and KEGG pathway analyses

Gene Ontology (GO) enrichment analysis for the source genes of differential circRNAs was performed by GOseq (v2.12) R packages based on the Wallenius non-central hyper-geometric distribution^28^. The KOBAS (v2.0) software was used to test the statistical enrichment of the circ-RNA host genes in the KEGG (Kyoto Encyclopedia of Genes and Genomes) pathways (http://www.genome.jp/keeg/)^29^.

### circRNA-miRNA interaction network prediction

The miRNA binding sites of *M. oryzae* circRNAs were predicted by the miRanda (v3.3a) software^19^ through alignment against miRNAs found in the filamentous fungi *Trichoderma reesei*^30^. The predicted interactions of seed-matching sequences between circRNAs and miRNAs were visualized by using Cytoscape (v3.4.0)^31^.

## Electronic supplementary material

Supporting information

Dataset S1

Dataset S2
