# Supplementary material for: Genome-wide Identification and characterization of circular RNAs in the rice blast fungus Magnaporthe oryzae
Source: Sci Rep. 2018 Apr 30;8:6757. doi: 10.1038/s41598-018-25242-w (PMC5928111; doi:10.1038/s41598-018-25242-w)
Supplement: Supplementary file 1 — Supporting information [file 41598_2018_25242_MOESM1_ESM.pdf]

Supplemental Materials

Genome-wide Identification and characterization of circular RNAs in the rice blast fungus *Magnaporthe oryzae*

Jialan Yuan[1,2], Zhao Wang[1], Junjie Xing[3], Qingyong Yang[2] & Xiao-Lin Chen[1,3]

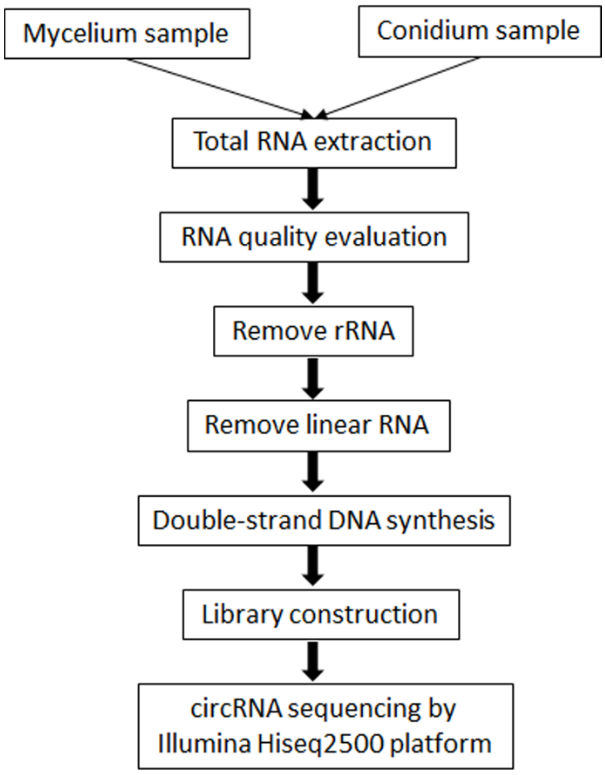

Fig. S1. Flowchart for identification of circRNAs in *M. oryzae*.

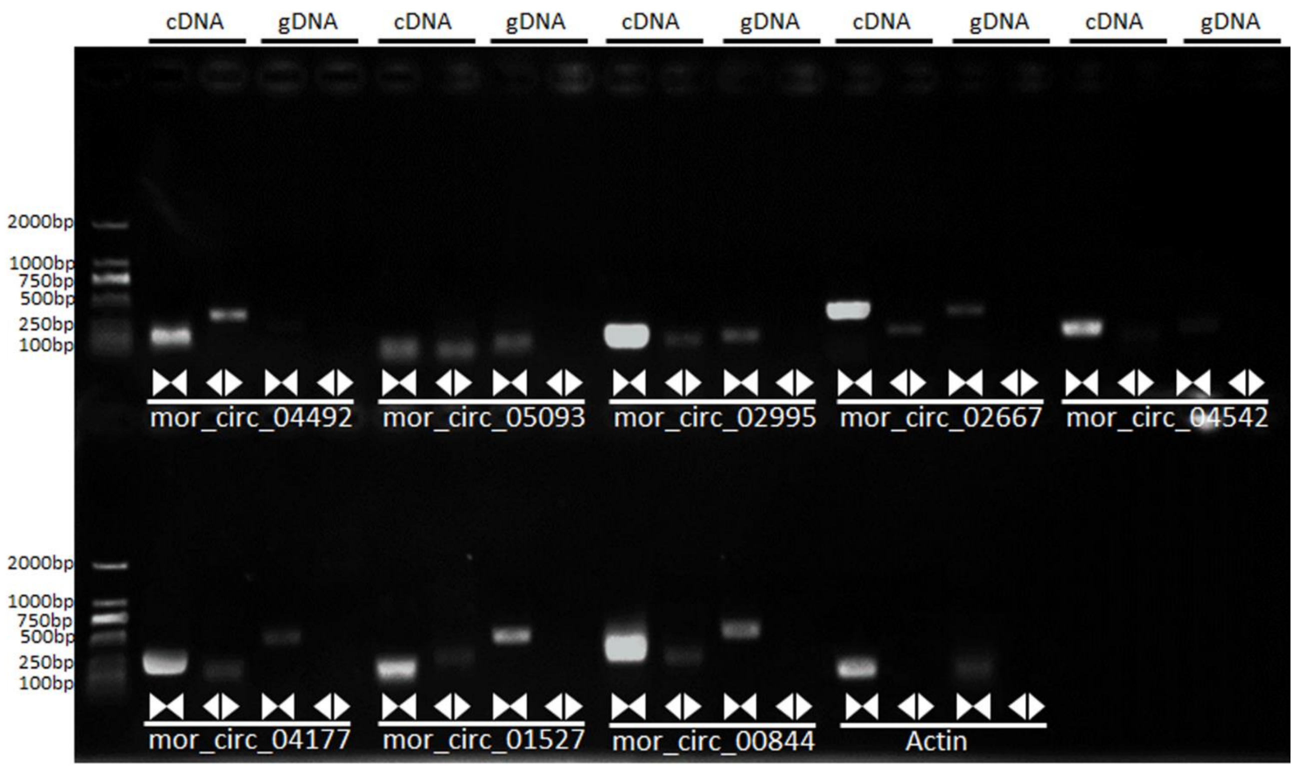

Fig. S2. Experimental validation of *M. oryzae* circRNAs. Divergent primers successfully amplified circRNAs in cDNA but failed in genomic DNA. Amplification for sequence of actin gene was used as a control.

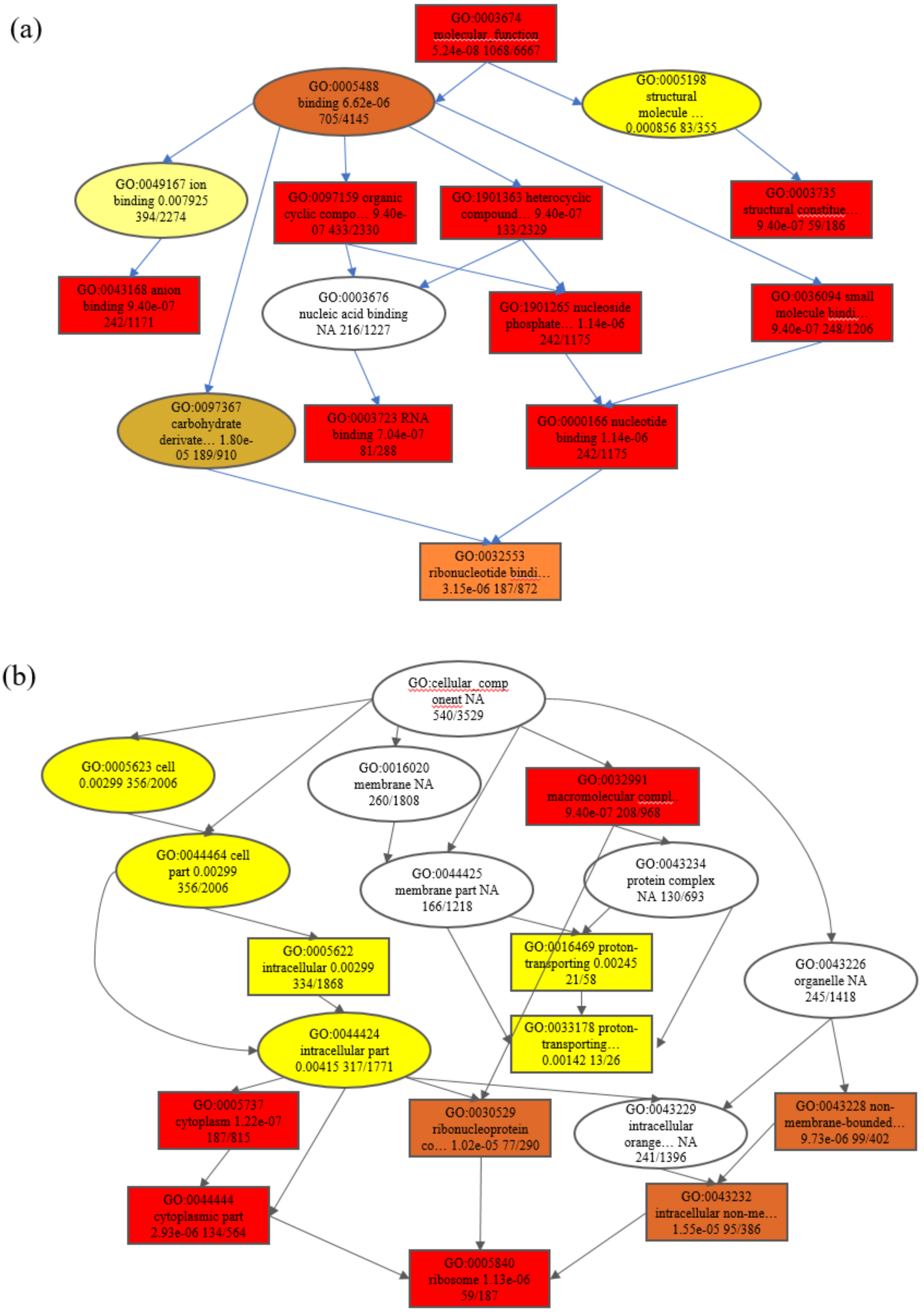

Fig. S3. The network of enriched GO terms of circRNAs-host genes in mycelium. (a) Significant GO terms of circRNA-host genes in the molecular function category (b) Significant GO terms of circRNA-host genes in the cellular component.

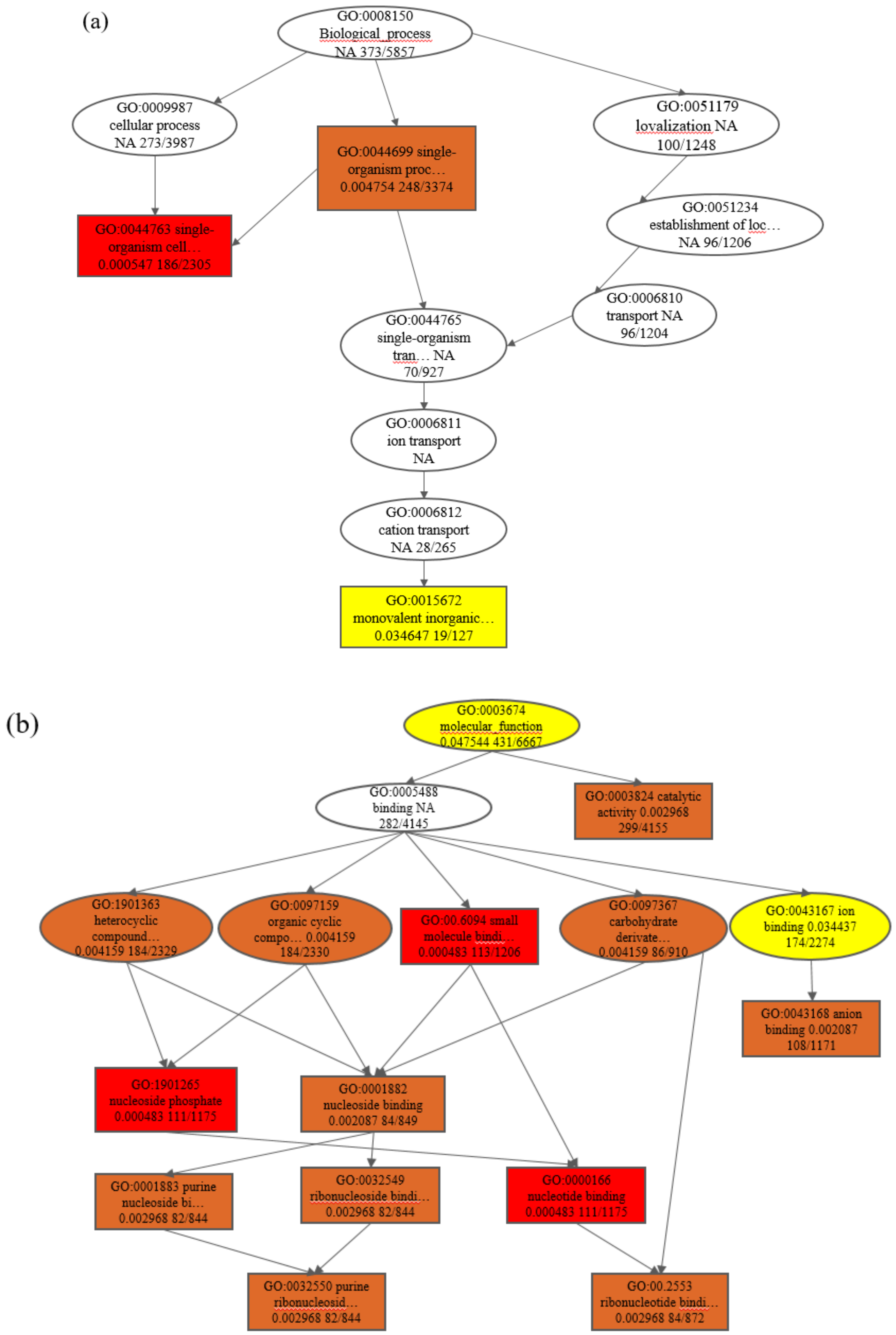

Fig. S4. The network of enriched GO terms of circRNAs-host genes in conidiim. (a) Significant GO terms of circRNA-host genes in the biological_process category (b) Significant GO terms of circRNA-host genes in the molecular_function category.

Table S1. Summary of RNA sequencing data analysis.

Sample	Raw Reads	Clean Reads	Error rate	Q20	Q30	GC content
CO_1_1	22,346,422	19,184,133	0.04	95.78	91.48	53.16
CO_1_2	22,346,422	19,184,133	0.04	94.10	88.97	53.00
CO_2_1	22,356,438	20,358,495	0.06	92.69	85.67	54.85
CO_2_2	22,356,438	20,358,495	0.05	92.41	86.35	54.59
MY_1_1	25,065,034	23,139,556	0.04	95.75	91.46	51.54
MY_1_2	25,065,034	23,139,556	0.04	93.93	88.74	51.32
MY_2_1	25,318,584	23,172,000	0.05	93.46	87.23	53.41
MY_2_2	25,318,584	23,172,000	0.05	92.50	86.75	53.06

Table S2. Distribution of circRNA in different scaffolds of *M. oryzae* strain P131.

Chr_name	circRNA_num		
P131_scaf1.3	1561	P131_scaf32.3	8
P131_scaf2.3	965	P131_scaf33.3	5
P131_scaf3.3	791	P131_scaf34.3	1
P131_scaf4.3	810	P131_scaf35.3	3
P131_scaf5.3	645	P131_scaf36.3	5
P131_scaf6.3	825	P131_scaf37.3	780
P131_scaf7.3	414	P131_scaf38.3	5
P131_scaf8.3	135	P131_scaf40.3	1
P131_scaf9.3	232	P131_scaf42.3	1
P131_scaf10.3	79	P131_scaf43.3	2
P131_scaf11.3	112	P131_scaf44.3	1
P131_scaf12.3	238	P131_scaf46.3	19
P131_scaf13.3	99	P131_scaf47.3	2
P131_scaf14.3	111	P131_scaf48.3	1
P131_scaf15.3	186	P131_scaf49.3	9
P131_scaf16.3	66	P131_scaf51.3	2
P131_scaf17.3	21	P131_scaf53.3	134
P131_scaf18.3	61	P131_scaf55.3	1
P131_scaf19.3	59	P131_scaf56.3	9
P131_scaf20.3	56	P131_scaf59.3	22
P131_scaf21.3	55	P131_scaf68.3	7
P131_scaf22.3	61	P131_scaf75.3	1
P131_scaf23.3	36	P131_scaf77.3	1
P131_scaf24.3	52	P131_scaf79.3	1
P131_scaf25.3	14	P131_scaf81.3	3
P131_scaf26.3	23	P131_scaf86.3	1
P131_scaf27.3	30	P131_scaf93.3	1
P131_scaf28.3	16	P131_scaf100.3	2
P131_scaf29.3	17	P131_scaf119.3	1
P131_scaf30.3	29	P131_scaf181.3	1
P131_scaf31.3	19		

Table S3. Convergent and divergent primers for validation of candidate circRNAs.

Primer	Primer sequence		cDNA	gDNA
4492convF	CAACAGGCTTCCGTTGGTGG	cDNA	231bp	325bp
4492convR	CGAGCTCCCTTCCACTCCAT			
4492divF	GAGATTGTCGACATGAGCAC	circDNA	222bp	
4492divR	CACCCCTCGAGGGGCTGGAT			
5093convF	GCTCGACACCACCACCGTCC	cDNA	214bp	214bp
5093convR	CGGTCGTAGGCTGCAGCGTG			
5093divF	GGCATCGAGGTCTTTACCAC	circDNA	172bp	
5093divR	CGTACCGCTCCAGGCTCAAC			
2995convF	CTCAACGATATCTTCGCTGG	cDNA	250bp	250bp
2995convR	AGCTCACGAACCCACTTGCG			
2995divF	GATGTCGGGAAGCAGGTCAT	circDNA	222bp	
2995divR	ATTCTGCGTGTCGAAGAAGT			
2667convF	CCCACATCTCTTGGCGCCTA	cDNA	419bp	419bp
2667convR	CACGAACTGCCTTTGCCTTC			
2667divF	GTCCGACTTTGACCTGGCAC	circDNA	198bp	
2667divR	TCGTATCCTCCTTGCCATAC			
4542convF	ATGGGTAAAGGAAAGCCTAG	cDNA	259bp	259bp
4542convR	CGAAAGCAGTAACCTTCTTG			
4542divF	TGGAGAAGGTCGGTGTTGAG	circDNA	198bp	
4542divR	GGCACGCTTCTTGTAGGCAA			
4177convF	ATACTCCAAGCCCAAGGAGT	cDNA	298bp	452bp
4177convR	GTTCTCACAGAGCAAAGACT			
4177divF	TTATCCCAGGACACGAGACC	circDNA	246bp	
4177divR	ACACCACACGCCTTGACCTT			
1527convF	AGGTCGAAAAGGCTATCGAT	cDNA	212bp	395bp
1527convR	GACAGGTTGGAGCCCTGATG			
1527divF	AGCCAACGACAACAGCCCTC	circDNA	257bp	
1527divR	TACCCACCATAGTCTAAACG			
0844convF	GCTTCGTATCCACGGGACTG	cDNA	248bp	407bp
0844convR	GCTCATGCTTGGTGTCGATG			
0844divF	TAGTGCTCTTCAGAGACTGG	circDNA	234bp	
0844divR	CAAGCAGCGTGTCCAAGACG			
3630convF	GGTCAGCAACGATTTGCTCC	cDNA	165bp	286bp
3630convR	CTTTGGCAGGATGGGCATGA			
3630divF	TTGGCATGCAGGGTCTCGGA	circDNA	228bp	
3630divR	CGACTCAACCCGGCAAACCA			
ActinconvF	GGACCGTATGCAGAAGGAGA	cDNA	109bp	109bp
ActinconvR	TGAGAATGGAACCACCGATC			
ActindivF	TCTCCTTCTGCATACGGTCC	circDNA		
ActindivR	GATCGGTGGTTCCATTCTCA			

Table S4. Top 20 enriched KEGG pathway in parental genes of mycelia circRNA.

Pathway term	Rich factor	Gene number	Qvalue
Carbon metabolism	0.565656566	56	0.078944769
Citrate cycle (TCA cycle)	0.807692308	21	0.100271923
Ribosome	0.514018692	55	0.134302066
Biosynthesis of amino acids	0.5	58	0.134302066
Glycolysis / Gluconeogenesis	0.564102564	22	0.589097544
Biosynthesis of secondary metabolites	0.393835616	115	0.757175202
Oxidative phosphorylation	0.465753425	34	0.780774001
2-Oxocarboxylic acid metabolism	0.514285714	18	0.946674754
Cysteine and methionine metabolism	0.5	19	0.946674754
Alanine, aspartate and glutamate metabolism	0.518518519	14	0.99927684
Pyruvate metabolism	0.484848485	16	0.99927684
Tryptophan metabolism	0.5	13	0.99927684
Phenylalanine metabolism	0.5	10	0.99927684
Pentose phosphate pathway	0.47826087	11	0.99927684
beta-Alanine metabolism	0.5	9	0.99927684
Valine, leucine and isoleucine degradation	0.461538462	12	0.99927684
Fatty acid degradation	0.5	8	0.99927684
Methane metabolism	0.44	11	0.99927684
RNA transport	0.377777778	34	0.99927684
Histidine metabolism	0.5	5	0.99927684

Table S5. Top 20 enriched KEGG pathway in parental genes of conidium circRNA.

Pathway term	Rich factor	Gene number	Qvalue
Cyanoamino acid metabolism	0.375	6	0.974950519
Glyoxylate and dicarboxylate metabolism	0.304347826	7	0.974950519
Tryptophan metabolism	0.269230769	7	0.974950519
Starch and sucrose metabolism	0.225	9	0.974950519
Oxidative phosphorylation	0.178082192	13	0.974950519
Protein processing in endoplasmic reticulum	0.175675676	13	0.974950519
Fatty acid biosynthesis	0.333333333	3	0.974950519
Biosynthesis of amino acids	0.155172414	18	0.974950519
RNA degradation	0.173913043	8	0.974950519
Glycolysis / Gluconeogenesis	0.179487179	7	0.974950519
Methane metabolism	0.2	5	0.974950519
Ether lipid metabolism	0.25	3	0.974950519
Alanine, aspartate and glutamate metabolism	0.185185185	5	0.974950519
MAPK signaling pathway - yeast	0.185185185	5	0.974950519
Nitrogen metabolism	0.214285714	3	0.974950519
Phagosome	0.166666667	6	0.974950519
Carbon metabolism	0.141414141	14	0.974950519
Arachidonic acid metabolism	0.25	2	0.974950519
Biosynthesis of secondary metabolites	0.126712329	37	0.974950519
Pentose phosphate pathway	0.173913043	4	0.974950519
